# Safety fears and relocation stressors related to flawed buildings: Ireland's defective concrete crisis

**DOI:** 10.1002/jts.70011

**Published:** 2025-09-02

**Authors:** Oisin Keenan, Jamie Murphy, Paul Dunlop, Eileen Doherty, Rachel McHugh, Karen Kirby

**Affiliations:** ^1^ Ulster University, School of Psychology Coleraine Northern Ireland; ^2^ Ulster University, School of Geography and Environmental Sciences Coleraine Northern Ireland; ^3^ Business School Ulster University Belfast Northern Ireland

## Abstract

The use of defective concrete in the construction of buildings in Ireland has led to widespread property deterioration, displacement, financial loss, and psychological distress for thousands of families. No research to date has examined mental health outcomes or associated risk factors among affected individuals. This study aimed to generate estimates of probable major depressive disorder (MDD), probable generalized anxiety disorder (GAD), probable posttraumatic stress disorder (PTSD), probable complex PTSD (CPTSD), and suicidal ideation in a sample of this population and to identify crisis‐related stressors associated with outcomes, while adjusting for trauma history, sociodemographic characteristics, and social support. A convenience sample of 393 adults completed a self‐report survey between March and September 2024. Estimates were 30.4% for MDD, 26.2% for GAD, 4.9% for PTSD, and 15.5% for CPTSD. Suicidal ideation, experienced after a property was suspected to have defective concrete, was present in 35.5% of the sample. Safety fears were associated with CPTSD, MDD, and suicidal ideation, odds ratios (*ORs*) = 2.09–4.39, whereas GAD was associated with relocating, *OR* = 2.25. These findings highlight the substantial psychological impact of the crisis and identify specific stressors associated with increased risk for adverse outcomes.

Since the early 2010s, thousands of homes and an unknown number of public buildings, schools, and businesses in western and northwestern Ireland have suffered from severe structural deterioration due to the use of defective concrete (DC) in their construction (Leemann et al., 2023). Governance failures in regulation and market surveillance enabled the use of deleterious materials (Cunningham, 2021; Doherty et al., 2022; Ó Broin, 2021), resulting in the gradual cracking and crumbling of buildings. Following years of campaigning, a government grant remediation scheme was introduced in 2020 and revised in 2023. However, the scheme has faced widespread criticism due to its complexity, delays, and insufficient financial coverage (Preece, 2024). Many affected individuals remain in unsafe, deteriorating properties, facing long‐term disruption, displacement, and uncertainty, with no clear path to resolution (Edwards, 2023).

Research on disaster‐exposed populations, where similar impacts occur, has consistently found elevated rates of depression, anxiety, posttraumatic stress disorder (PTSD), and suicidal ideation (Karimi et al., 2022; Keya et al., 2023). Outcomes are influenced by factors such as gender, relocation, financial adversity, housing tenure, property damage, social support, and high levels of postevent stressors (Galea et al., 2008; Keya et al., 2023; Li et al., 2024). Given the chronic nature of Ireland's defective concrete crisis (DCC), with homeowners living in deteriorating conditions for years before government action, this stressor may align more closely with pathways leading to complex PTSD (CPTSD), which is particularly associated with prolonged or repeated trauma, often in situations where resolution appears difficult (Brewin et al., 2017; Maercker et al., 2022).

Trauma history complicates investigations of traumatic stress, as individuals who have experienced potentially traumatic events (PTEs) may be more vulnerable to subsequent stressors (Matosin et al., 2017). Cumulative trauma exposure has also been associated with an increased risk of PTSD and CPTSD (Frost et al., 2019). Accounting for trauma history is, therefore, critical to attaining an accurate understanding of mental health outcomes following a particular event.

Despite growing awareness, no research to date has examined mental health outcomes or their predictors among this population. Given widespread reports of hardship and the mental health consequences of unsafe housing, disrupted living, and displacement, there is a clear need to investigate the mental health status of this unstudied population (Andrews et al., 2024; Edwards, 2023; Morina et al., 2018). This study aimed to generate estimates of depression, anxiety, posttraumatic stress symptoms, and suicidal ideation and to identify DCC‐related stressors predicting these outcomes, while controlling for trauma history, sociodemographic characteristics, and social support.

## METHOD

### Participants and procedure

This study used data from a cross‐sectional Qualtrics survey conducted between March 4, 2024, and September 20, 2024. Ethical approval was granted by Ulster University's research ethics committee (Study No. FCPSY‐24‐002‐A). Although no official registry of affected homeowners exists, prior research estimates that at least 5,000 households have been impacted (Leemann et al., 2023). Given the absence of a registry and the difficulty accessing this hard‐to‐reach population, many of whom have lived without formal support or recognition for years, a representative sampling frame was not attainable. Crisis campaign and community groups (i.e., the Mica Action Group and the Inishowen Development Partnership), therefore, afforded an alternative sampling strategy. Using convenience sampling, participants were recruited through these groups using social media and membership contacts. Although representativeness could not be formally assessed, the geographic distribution of respondents based in County Donegal (93.1% of the sample) closely mirrored official figures for grant scheme applications through the Donegal County Council (2024; see Supplementary Figure S1).

Eligible participants were adults (aged 18 years or older) who lived in or owned a property affected by DC, excluding individuals with serious cognitive impairments (e.g., Alzheimer's disease). Informed consent was obtained following a briefing on study aims, confidentiality, risks, and withdrawal rights. Mental health and DCC‐related support resources were also provided. Table 1 presents the sample's sociodemographic characteristics (*N* = 393).

**TABLE 1:** Sociodemographic characteristics of the sample

Variable	*n*	%
Gender		
Male	128	32.6
Female	263	66.9
Transgender, nonbinary, or prefer not to say	2	0.5
Age (years)		
18–24	10	2.5
25–34	7	1.8
35–44	95	24.2
45–54	175	44.5
55–64	76	19.3
≥ 65	30	7.6
County		
Donegal	364	93.1
Mayo	13	3.3
Clare	5	1.3
Limerick	2	0.5
Other	7	1.8
Area of residence		
Rural	274	69.9
Town	79	20.2
Suburb	33	8.4
City	6	1.5
Post secondary qualification		
Yes	311	79.1
No	82	20.9
Married		
Yes	316	80.4
No	77	19.6
Living alone		
Yes	28	7.1
No	365	92.9
Children in household		
Yes	256	65.1
No	137	34.9
Household income (EUR)		
€0–19,999	31	8.1
€20,000–39,999	94	24.5
€40,000–59,999	86	22.5
€60,000–79,999	72	18.8
€80,000‐99,999	43	11.2
€100,000‐119,999	30	7.8
≥ €120,000	27	7.0
Housing tenure (of defective concrete‐affected home)		
Own outright	126	38.5
Own with mortgage	190	58.1
Other (e.g., renting or living rent free)	11	3.4
Employment status		
Full‐time	239	60.8
Part‐time	71	18.1
Retired	35	8.9
Other (e.g., student or temporary employment)	48	12.2
Caregiver		
Yes	93	23.7
No	300	76.3
Previous trauma exposure		
Yes	227	69.6
No	99	30.4
Number of potentially traumatic events experienced		
1	75	23.0
2	63	19.3
≥ 3	89	27.3

### Measures

#### *ICD‐11* PTSD and CPTSD

The 18‐item International Trauma Questionnaire (ITQ; Cloitre et al., 2018) assessed symptoms related to the DCC. Six items measure PTSD symptoms across three clusters (reexperiencing, avoidance, and sense of current threat), and six items measure disturbances in self‐organization (DSO; i.e., affect dysregulation, negative self‐concept, and disturbances in relationships), each followed by three functional impairment (FI) items. The measure is based on criteria outlined in the *International Statistical Classification of Diseases and Related Health Problems* (11th ed.; *ICD‐11*; World Health Organization, 2019). Items are rated on a scale of 0 (*not at all*) to 4 (*extremely*), with scores of 2 or higher indicating endorsement. A probable PTSD diagnosis requires the endorsement of one symptom per PTSD cluster and one PTSD FI item. A probable CPTSD diagnosis requires meeting the PTSD criteria plus the endorsement of one symptom per DSO cluster and one DSO FI item. Individuals can meet the criteria for PTSD or CPTSD, but not both. The ITQ has shown strong psychometric properties (McGinty et al., 2024), and internal reliability was high for both the PTSD, Cronbach's α = .89, and DSO, Cronbach's α = .92, subscales in the present sample.

##### Depressive symptoms

The Patient Health Questionnaire–9 (PHQ‐9; Kroenke & Spitzer, 2002) was used to assess probable major depressive disorder (MDD). Participants were asked to rate nine symptoms on a scale of 0 *(not at all*) to 3 (*nearly every day*) regarding how often they experienced each symptom over the past 2 weeks. Total scores range from 0 to 27, with cutoff thresholds of 10 or 15 recommended to indicate probable MDD (Hyland, Vallières, et al., 2022); this study used the stricter cutoff of 15. The PHQ‐9 has strong psychometric support (Beard et al., 2016), and internal reliability was excellent, Cronbach's α = .90, in the present sample.

##### Anxiety symptoms

The seven‐item Generalized Anxiety Disorder scale (GAD‐7, Spitzer et al., 2006) was used to assess probable GAD. Participants rated symptoms over the past two weeks on a scale of 0 (*not at all*) to 3 (*nearly every day*). Total scores range from 0 to 21, with cutoff thresholds of either 10 or 15 recommended to indicate probable GAD (Hyland, Vallières, et al., 2022); this study used the stricter cut‐off of 15 or higher. The GAD‐7 has strong psychometric support (Johnson et al., 2019), and internal reliability was excellent, Cronbach's α = .92, in this sample.

##### Suicidal ideation

One item adapted from the 2014 English Adult Psychiatric Morbidity Survey (McManus et al., 2016) was used to assess suicidal ideation since participants first suspected their property was affected. Responses were scored as 1 for “yes” or 0 for “no.” This item was part of an optional survey section. Of 389 respondents, 297 (76.3%) opted in, and 293 valid responses were included.

##### Covariates

###### Sociodemographic characteristics

Participants reported their gender, age group, and household income. For regression analyses, age was recoded into five categories (combining 18–24 years and 25–34 years), children in the household as present (1) or not (0), housing tenure as own outright (0) or mortgage/other (1), and caregiver status as yes (1) or no (0).

###### DCC‐related stressors

As this is a previously unstudied population, a checklist was developed based on media reports, public testimony, and consultation with campaign and community groups to capture common crisis‐related stressors (see Supplementary Material). Participants indicated whether they had experienced and/or found stressful each of the following: relocating, safety fears in one's own home, navigating the legal or administrative challenges of testing and registering on schemes, financial stress or impact, mental health challenges, impact on family members/household, lack of adequate support services, lack of adequate governmental response, other (with a prompt to specify), and none. For the regression analyses, mental health challenges, other, and none were excluded. Items were coded as “yes” (1) or “no” (0).

###### Social support

The 12‐item Multidimensional Scale of Perceived Social Support (MSPSS; Zimet et al., 1988) was used to assess perceived support from family, friends, and significant others. Items were rated on a scale of 1 (*very strongly disagree*) to 7 (*very strongly agree*). A total mean score was computed and recoded as low (1.0–2.9), moderate (3.0–5.0), or high (5.1–7.0) support (Zimet, 2016). The MSPSS has shown good psychometric properties (Zimet et al., 1990). Internal reliability of MSPSS scores was excellent in this sample, Cronbach's α = .94.

###### Trauma history

The Life Events Checklist for *DSM‐5* (LEC‐5; Weathers et al., 2013) was used to assess exposure to 16 PTEs and one additional item (“any other stressful life event”). Response options include “happened to me,” “witnessed it,” “learned about it,” “part of my job,” “not sure,” and “doesn't apply,” with multiple choices allowed. Only items endorsed as “happened to me” were coded as 1, with all others 0, consistent with previous research using a strict definition of trauma exposure (Nesterko et al., 2020). The additional item was excluded, as it could be attributed to this crisis. A count variable was computed and recoded as exposure to 0, 1, 2, or 3 or more (≥ 3) PTEs.

### Data analysis

Estimates were calculated for MDD, GAD, PTSD, CPTSD, and suicidal ideation. Separate binary logistic regressions were conducted to identify predictors of mental health outcomes. All models included sociodemographic variables (age, gender, income, children in household, housing tenure, caregiver status), social support, trauma history, and DCC‐related stressors (relocating, safety fears, navigating administrative processes, financial stress/impact, familial impact, lack of support services, and government response). Associations were reported as odds ratios (*ORs*) with 95% confidence intervals (CIs). Listwise deletion was used to handle missing data, which were minimal and appeared to be missing at random. Analyses were conducted using SPSS (Version 29).

## RESULTS

### Mental health estimates

Nearly one third of participants (30.4%, *n* = 119) scored above the threshold for probable MDD, and 26.2% (*n* = 99) scored above the threshold for probable GAD.

Probable PTSD and CPTSD were present in 4.9% (*n* = 18) and 15.5% (*n* = 57) of the sample, with 20.4% (*n* = 75) of participants meeting the criteria for either condition. Of the 293 valid responses for the item related to suicidal ideation, 35.5% (*n* = 104) participants reported experiencing suicidal ideation since suspecting their property was affected by DC.

### Regression analyses

#### Stressors

Table 2 presents binary logistic regression results. After adjusting for sociodemographic characteristics, social support, and trauma history, “safety fears” was significantly associated with probable CPTSD, *OR* = 4.39, 95% CI [1.54, 12.52]; probable MDD, *OR* = 2.09, 95% CI [1.07, 4.08]; and suicidal ideation, *OR* = 2.36, 95% CI [1.12, 5.05]. Participants who reported safety fears had 4.4‐fold higher odds of meeting the criteria for CPTSD, 2.1‐fold higher odds of MDD, and 2.4‐fold higher odds of suicidal ideation. “Relocating” was associated with significantly higher odds of probable GAD, *OR* = 2.25, 95% CI [1.22, 4.16].

**TABLE 2:** Binary logistic regression associations between identified crisis‐related stressors and mental health outcomes, adjusted for sociodemographic characteristics, social support, and other trauma exposure

	CPTSD (*n =* 294)		Depression (*n* = 293)		Anxiety (*n =* 294)		Suicidal Ideation (*n* = 237)	
Variable	*OR*	95% CI	*OR*	95% CI	*OR*	95% CI	*OR*	95% CI
Stressors								
Relocating	1.200	[0.580, 2.481]	1.521	[0.848, 2.728]	2.250^*^	[1.218, 4.158]	1.076	[0.556, 2.081]
Safety fears	4.389	[1.539, 12.518]	2.088^*^	[1.069, 4.077]	1.724	[0.867, 3.428]	2.362^*^	[1.105, 5.048]
Paperwork	0.720	[0.241, 2.148]	0.689	[0.303, 1.566]	0.791	[0.338, 1.847]	1.776	[0.659, 4.787]
Financial	1.620	[0.256, 10.263]	1.382	[0.368, 5.197]	4.089	[0.821, 20.365]	4.170	[0.767, 22.676]
Familial	1.744	[0.546, 5.572]	1.613	[0.669, 3.892]	0.695	[0.297, 1.629]	0.958	[0.358, 2.559]
Lack of support	0.947	[0.385, 2.332]	1.289	[0.632, 2.630]	1.265	[0.606, 2.643]	1.334	[0.579, 3.073]
Government	2.255	[0.236, 21.565]	1.203	[0.321, 4.506]	0.869	[0.235, 3.208]	0.410	[0.104, 1.612]
Sociodemographic characteristics								
Age	1.073	[0.682, 1.687]	0.953	[0.661, 1.374]	0.694	[0.477, 1.008]	0.989	[0.651, 1.501]
Gender	1.031	[0.498, 2.134]	0.996	[0.560, 1.773]	1.438	[0.779, 2.654]	0.888	[0.463, 1.705]
Income	0.868	[0.686, 1.098]	0.868	[0.723, 1.043]	0.942	[0.782, 1.135]	0.979	[0.801, 1.197]
Dependents	0.568	[0.241, 1.339]	0.847	[0.425, 1.690]	0.439^*^	[0.215, 0.895]	1.213	[0.563, 2.610]
Tenure	3.111^*^	[1.307, 7.405]	1.951^*^	[1.030, 3.699]	1.098	[0.578, 2.085]	1.473	[0.707, 3.069]
Caregiver	1.314	[0.605, 2.854]	2.020^*^	[1.086, 3.758]	1.262	[0.661, 2.408]	2.006	[0.968, 4.155]
Social support	0.379^***^	[0.227, 0.632]	0.475^***^	[0.309, 0.730]	0.714	[0.461, 1.105]	0.365^***^	[0.221, 0.603]
1 PTE	1.634	[0.577, 4.630]	1.143	[0.534, 2.448]	1.589	[0.725, 3.482]	1.326	[0.539, 3.260]
2 PTE	1.374	[0.475, 3.976]	1.253	[0.565, 2.777]	1.036	[0.435, 2.465]	1.568	[0.651, 3.780]
3 PTE	2.855^*^	[1.117, 7.299]	1.452	[0.709, 2.970]	2.500^*^	[1.183, 5.283]	2.919	[1.303, 6.539]

*Note*: CPTSD = complex posttraumatic stress disorder; *OR* = odds ratio; 95% CI = 95% confidence interval; PTE = potentially traumatic event.

* *p* < .05. **p < .01. ***p < .001.

Although not statistically significant, “financial stress/impact” showed large associations with probable GAD, *OR* = 4.09, 95% CI [0.82, 20.37], and suicidal ideation, *OR* = 4.17, 95% CI [0.77, 22.68]. These findings suggest potential relevance in larger samples but should be interpreted with caution due to wide confidence intervals.

### Other risk factors

Not owning one's home outright (e.g., mortgaging or renting) was associated with probable CPTSD, *OR* = 3.11, 95% CI [1.31, 7.41], and MDD, *OR* = 1.95, 95% CI [1.03, 3.70]. Caregiving was associated with probable MDD, *OR* = 2.02, 95% CI [1.09, 3.76], whereas not having children in the household was associated with probable GAD, *OR* = 0.44, 95% CI [0.22, 0.90].

Previous exposure to three or more PTEs was associated with probable CPTSD, *OR* = 2.86, 95% CI [1.12, 7.30]; probable GAD, *OR* = 2.50, 95% CI [1.18, 5.28]; and suicidal ideation, *OR* = 2.92, 95% CI [1.30, 6.54]. Higher social support was associated with lower odds of probable CPTSD, *OR* = 0.38, 95% CI [0.23, 0.63]; probable MDD, *OR* = 0.48, 95% CI [0.31, 0.73]; and suicidal ideation, *OR* = 0.37, 95% CI [0.22, 0.60].

## DISCUSSION

Although substantial evidence exists regarding the mental health consequences of natural disasters and other large‐scale PTEs, this study is the first to examine the unique context of gradual property deterioration in Ireland. Probable PTSD (4.9%) and CPTSD (15.5%) estimates were approximately double recent Irish general population estimates (2.4% and 8.8%, respectively; McGinty et al., 2024). CPTSD was over three times more common than PTSD, consistent with its association with prolonged trauma exposure (Brewin et al., 2017; Hyland et al., 2017). The chronic stress of living in deteriorating homes, amid ongoing uncertainty, financial strain, and administrative barriers, reflects a unique type of stressor. Rather than a past traumatic experience, individuals face a persistent and unresolved threat. This prolonged exposure to potential harm, without clear resolution, may contribute to complex trauma responses (Frost et al., 2019; Hyland et al., 2017).

The proportion of participants who scored above the clinical threshold for probable MDD (30.4%) and probable GAD (26.2%) were nearly three‐ and four‐times higher, respectively, than general population estimates (11.5% and 7.1%; Hyland, Vallières, et al., 2022), based on the same PHQ‐9 and GAD‐7 cutoffs (i.e., 15). When applying the more commonly used cutoff of 10 for moderate symptom severity, clinically significant depression and anxiety figures in this sample increase to 54.7% (*n* = 214) and 50.0% (*n* = 189), which are, again, more than double national estimates (22.8% and 20.0%, respectively; Hyland et al., 2020). Over one third of respondents reported suicidal ideation (35.5%), exceeding the Irish lifetime estimate of 29.5% (Hyland, Rochford, et al., 2022). Notably, these Irish comparison estimates may be somewhat elevated due to their time of study (the COVID‐19 pandemic). Furthermore, the current findings are comparable to rates reported in meta‐analyses and pooled prevalence studies of individuals affected by disasters and displacement, including refugees and populations exposed to earthquakes or floods (Blackmore et al., 2020; Cénat et al., 2020; Cruz et al., 2020; Dai et al., 2016; Golitaleb et al., 2022; Lechner‐Meichsner et al., 2024).

“Safety fears” was associated with significantly higher odds of CPTSD, probable MDD, and suicidal ideation and represented the strongest crisis‐related stressor across outcomes. This finding may reflect the role of perceived threat in perpetuating the emotional dysregulation and hypervigilance central to traumatic stress responses (Brewin et al., 2017; Putica & Agathos, 2024). For those living in crumbling homes, the persistent sense of an unsafe environment may chronically activate the stress response, hinder recovery, and maintain a heightened state of arousal (Matosin et al., 2017; Putica & Agathos, 2024). “Relocating” was associated with probable GAD, likely reflecting concerns associated with displacement, such as securing alternative accommodation and disrupted routines. Extensive literature supports the association between displacement and anxiety (Morina et al., 2018). DCC stressors were assessed in the context of prior trauma. Notably, the effects of safety fears and relocating are comparable to or stronger than exposure to three or more lifetime PTEs.

Additional risk factors include caregiving status, which was associated with depression, and insecure housing tenure (e.g., mortgaging or renting), which was associated with both CPTSD and depression, consistent with research linking tenure insecurity to psychological distress (Li et al., 2024). Social support showed a protective effect for CPTSD, probable MDD, and suicidal ideation but not for probable GAD. No significant effects were found for age, gender, or income.

This study has several strengths, including the use of standardized measures, stricter PHQ‐9 and GAD‐7 cutoffs, and analyses that controlled for trauma history and social support. There are also several limitations to discuss, including a nonprobability sample, predominantly female participants (66.9%), and a risk of potential response bias (i.e., participants most affected by the crisis may have been more likely to participate, whereas those least affected may have refrained). The suicidal ideation item was optional and not all participants responded, which may introduce further bias. Data were self‐reported, and the cross‐sectional design prevents causal conclusions. Although stricter cutoffs improve specificity, survey data lack the precision of structured interviews. Although adjusting for prior trauma exposure indicates that mental health outcomes are independent of extant trauma history, it is possible these PTEs occurred after DCC exposure began. Other potentially important variables, such as preexisting mental health conditions unrelated to trauma and personality traits (e.g., neuroticism), were not assessed.

Despite its limitations, the study offers novel insights into an understudied population and identifies crisis‐specific predictors of distress that warrant further investigation. The findings indicate a substantial mental health burden among affected individuals, with levels markedly above Irish general population estimates. Safety fears and relocating emerged as key stressors associated with adverse outcomes, even after accounting for sociodemographic characteristics, social support, and trauma history.

## AUTHOR NOTE

This research was funded by the Northern Ireland Department for the Economy.

## OPEN PRACTICES STATEMENT

Research data are not shared, as further analysis is being conducted as part of a PhD project; data will be available upon request after the project's completion.

## Supporting information



Supporting‐Information
