# Supplementary material for: Safety fears and relocation stressors related to flawed buildings: Ireland's defective concrete crisis
Source: J Trauma Stress. 2025 Sep 2;39(1):143–50. doi: 10.1002/jts.70011 (PMC12890771; doi:10.1002/jts.70011)
Supplement: Supplementary file 1 — Supporting‐Information [file JTS-39-143-s001.docx]

**Appendix A**

**FIGURE 1**

*Proportion of Donegal participants by municipal district compared to those who applied to the remediation grant scheme through Donegal County Council*

**Appendix B**

*Sources for defective concrete crisis-related stressor questionnaire*

Bradley, C., & Darwish, M. (2022, October 15). ‘People are suicidal, I’ve considered it myself’ – Mica homeowners on the brink*. Irish Mirror*. <https://www.irishmirror.ie/news/people-suicidal-ive-considered-myself-28239829>

Crumbling house grant scheme must improve – report. (2024, January 19). *BBC News*. <https://www.bbc.co.uk/news/articles/crglejqqe88o>

Cunningham, P. (2021, June 16). Final bill for mica damage may exceed €1.5bn - O'Brien. *RTÉ News.* <https://www.rte.ie/news/politics/2021/0615/1228271-mica-leaders-questions/>

Doherty, E., Carcary, M., Ramsey, E., Kelly, D., & Dunlop, P. (2022). An examination of governance failure by the Irish State: The critical case of the ‘Mica’/defective blocks issue. Proceedings of the *European Conference on Management Leadership and Governance*, *18*(1), 154–163. <https://doi.org/10.34190/ecmlg.18.1.934>

Donegal residents fear for health and children's safety in substandard Mica homes with no help in sight. (2023, February 5). *Irish Independent.* <https://www.independent.ie/irish-news/donegal-residents-fear-for-health-and-childrens-safety-in-substandard-mica-homes-with-no-help-in-sight/42328364.html>

Edwards, R. (2023, June 11). ‘Am I going to be homeless?’ – Mica wounds deepen as crisis takes heavy toll on homes and health of residents. *Irish Independent.* <https://www.independent.ie/irish-news/am-i-going-to-be-homeless-mica-wounds-deepen-as-crisis-takes-heavy-toll-on-homes-and-health-of-residents/a851228926.html>

Glenn, L. (2021, July 3). MICA homes in Donegal: Our children have been ‘robbed of their childhood’. *Derry Journal*. <https://www.derryjournal.com/news/environment/mica-homes-in-donegal-our-children-have-been-robbed-of-their-childhood-3295378>

Glenn, L. (2023, January 16). Mica Action Group is urging vulnerable homeowners in need of extra support to get in touch. *Derry Journal*. <https://www.derryjournal.com/news/environment/mica-action-group-is-urging-vulnerable-homeowners-in-need-of-extra-support-to-get-in-touch-3989099>

Hennessy, M. (2022, February 21). 'The anxiety was unbelievable': Mica homeowners feared for their safety during Storm Franklin. *The Journal.* <https://www.thejournal.ie/mica-donegal-storms-5688980-Feb2022/>

Matthews, J. (2023, July 13). Mica homeowners have 'outstanding concerns' about new scheme, committee told. *The Journal.* <https://www.thejournal.ie/mica-victims-outline-concerns-about-new-redress-scheme-6117500-Jul2023/>

McBride, M. (2024, January 23). Battling storms living in crumbling mica homes. *BBC News.* <https://www.bbc.co.uk/news/articles/cg3k70w7ezpo>

McCurry, C. (2021, October 7). Mica crisis: ‘My house is failing again. I can't sell it and I flushed my hard-earned money down the toilet’. *Irish Independent*. <https://www.independent.ie/irish-news/politics/mica-crisis-my-house-is-failing-again-i-cant-sell-it-and-i-flushed-my-hard-earned-money-down-the-toilet/40927526.html>

McCurry, C. (2021 October 8). ‘People are afraid their homes will collapse as they sleep at night’. *Irish Independent*. <https://www.independent.ie/irish-news/people-are-afraid-their-homes-will-collapse-as-they-sleep-at-night/40928551.html>

McDaid, B. (2021, June 1). MICA homes in Donegal: ‘They can’t let it go like this; houses are literally falling down’. *Derry Journal.* <https://www.derryjournal.com/news/environment/mica-homes-in-donegal-they-cant-let-it-go-like-this-houses-are-literally-falling-down-3257419>

McLaughlin, R. (2021, August 3). Mica crisis could raise homeless rates beyond imagination, charity warns. *Donegal Daily.* <https://www.donegaldaily.com/2021/08/03/mica-crisis-could-raise-homeless-rates-beyond-imagination-charity-warns-2/>

Mica: Donegal teens speak out about crippling fears. (2021, July 21). *Donegal Daily.* <https://www.donegaldaily.com/2021/07/21/mica-donegal-teens-speak-out-about-crippling-fears/>

O’Reilly, R. (2023, September 7). Mould and debt add to homeowners' mica and pyrite nightmares. *RTÉ News.* <https://www.rte.ie/news/primetime/2023/0905/1403578-mould-and-debt-add-to-homeowners-mica-and-pyrite-nightmares/>

Quinn, T. (2021, June 18). Ireland’s mica scandal causing ‘mental health tsunami’ as homes crumble in front of families’ eyes. *Irish Mirror*. <https://www.irishmirror.ie/news/irish-news/irelands-mica-scandal-causing-mental-24349860>

Survey reveals impact on health of dealing with mica. (2021, September 16). *Donegal News.* <https://donegalnews.com/survey-reveals-impact-on-health-of-dealing-with-mica/>
